# Feeling Older and Constrained: Synergistic Influences of Control Beliefs and Subjective Age on Cognition

**DOI:** 10.1093/geroni/igaa057.1246

**Published:** 2020-12-16

**Authors:** Eric Cerino, Erica O’Brien, David Almeida

**Affiliations:** Pennsylvania State University, University Park, Pennsylvania, United States

## Abstract

Control beliefs are important correlates of cognitive health and aging. In addition, how old or young one feels is a self-perception of aging that may play a role in understanding control-cognition associations. We explored whether subjective age moderates associations among control beliefs and cognitive performance using data from the third wave of the national Midlife in the United States study. The analytic sample comprised of 2,621 adults aged 39–93 (Mage=64.06, SD=11.15; 55.51% female) that completed measures of control (mastery, perceived constraints), subjective age (how old you feel most of the time), and cognition (executive function, episodic memory) via telephone administration. Hierarchical regression analyses were conducted to examine whether mastery, perceived constraints, and subjective age were associated with cognitive performance, adjusting for chronological age, gender, education, marital status, and self-rated health. For executive function, there was a significant perceived constraints by subjective age interaction. Higher levels of perceived constraints were associated with worse executive function (Est.=-0.05, SE=0.01, p<.001), and this association was amplified among those with relatively older subjective ages (Est.-0.10, SE=0.02, p<.001). For episodic memory, higher levels of perceived constraints were associated with worse performance (Est.=-0.07, SE=0.03, p<.001), while reporting a more youthful subjective age was associated with better performance (Est.-0.10, SE=0.02, p<.001). Mastery was not associated with either cognitive domain (ps>.05). Results suggest that perceiving constraints in life may confer greatest risk to cognitive performance among adults who feel older than their actual age, whereas perceiving a more youthful subjective age may be more facilitative.

